# Long-Term Major Adverse Kidney and Cardiovascular Events in Predialysis Chronic Kidney Disease: A Real-World Cohort Study

**DOI:** 10.3390/medicina62101957

**Published:** 2026-10-10

**Authors:** Ayşe Karaduru Avcı, Ayca Inci, Rauf Avcı, Uğur Büyükokudan, Fatih Sargin, Lütfullah Zahit Koç, Üstün Yılmaz

**Affiliations:** 1 Department of Internal Medicine, The Antalya City Hospital, 07080 Antalya, Turkey; sarginfatih@gmail.com; 2 Nephrology Department, Faculty of Medicine, Akdeniz University, 07058 Antalya, Turkey; aycainci@akdeniz.edu.tr; 3 Cardiology Department, Antalya Training and Research Hospital, 07100 Antalya, Turkey; drraufavci07@gmail.com; 4 Department of Public Health, Faculty of Medicine, Afyonkarahisar Health Sciences University, 03030 Afyonkarahisar, Turkey; u_buyukokudan@hotmail.com; 5 Internal Medicine Department, Antalya Training and Research Hospital, 07100 Antalya, Turkey; lutfullahkoc@gmail.com; 6 Nephrology Department, Antalya Training and Research Hospital, 07100 Antalya, Turkey; ustunfy@gmail.com

**Keywords:** chronic kidney disease, predialysis, major adverse kidney event, major adverse cardiovascular event, albuminuria, SGLT2 inhibitor

## Abstract

*Background and Objectives:* Long-term renal and cardiovascular outcomes represent major clinical burdens in predialysis chronic kidney disease (CKD). This study aimed to evaluate the long-term incidence of and clinical factors associated with major adverse kidney events (MAKEs) and incident major adverse cardiovascular events (MACEs) in patients with predialysis CKD. *Materials and Methods:* This retrospective cohort included 263 adults with predialysis CKD followed for at least five years. Patients with baseline dialysis, kidney transplantation, coronary artery disease, heart failure, stroke, or malignancy were excluded. MAKE was defined as chronic dialysis, kidney transplantation, or a ≥50% decline in baseline eGFR. MACE comprised nonfatal myocardial infarction, nonfatal stroke, or cardiovascular death. Separate multivariable Cox regression models were constructed for MAKE and MACE, with treatment exposures modeled as time-dependent covariates. Fine–Gray competing-risk regression was performed as a sensitivity analysis. *Results:* During a median follow-up of 2829 days, MAKE occurred in 97 patients (36.9%) and MACE in 53 (20.2%), corresponding to incidence rates of 47.8 and 26.0 per 1000 person-years, respectively. In multivariable Cox models for MAKE, lower baseline eGFR (HR per 10 mL/min/1.73 m^2^ increase 0.84, 95% CI 0.72–0.98; *p* = 0.024) and greater albuminuria severity were independently associated with risk. Compared with normal albuminuria, microalbuminuria and macroalbuminuria were associated with approximately fourfold and eightfold higher hazards, respectively (HR 4.15, 95% CI 1.91–9.00; and HR 7.73, 95% CI 4.01–14.90; both *p* < 0.001). For MACE, older age (HR per 10 years 1.52, 95% CI 1.12–2.06; *p* = 0.007) and diabetes mellitus (HR 3.00, 95% CI 1.60–5.62; *p* < 0.001) were independently associated with higher risk, while higher baseline eGFR was associated with lower risk in the treatment-adjusted models (HR per 10 mL/min/1.73 m^2^ 0.76; *p* = 0.032–0.037). Neither time-dependent SGLT2 inhibitor nor ACE inhibitor/ARB use was significantly associated with MAKE or MACE. Fine–Gray competing-risk analyses yielded broadly consistent results. SGLT2 inhibitor exposure occurred in 75 patients (28.5%), and treatment-related findings should be interpreted cautiously given the limited exposure and observational design. *Conclusions:* MAKE and MACE were common in predialysis CKD. Lower baseline kidney function and albuminuria severity were associated with adverse kidney outcomes, whereas older age, diabetes mellitus, and lower baseline kidney function were associated with incident cardiovascular events. These findings highlight distinct renal and cardiovascular risk profiles in predialysis CKD.

## 1. Introduction

Chronic kidney disease (CKD) is a major global health problem associated not only with progressive loss of kidney function but also with substantial cardiovascular morbidity, mortality, and healthcare burden [1]. According to the Global Burden of Disease 2021 estimates, CKD ranks 11th among the causes of death worldwide, and projections of future disease burden suggest that it could rise to 5th place by 2050 [2]. Therefore, CKD is recognized as a major public health priority because of its adverse clinical outcomes and increasing burden on healthcare systems [3].

The risk of cardiovascular disease is markedly increased in the CKD population. This risk extends beyond atherosclerotic events and includes myocardial infarction, structural heart disease, arrhythmias, and sudden cardiac death. Moreover, in a significant proportion of individuals with CKD, the risk of death from cardiovascular disease exceeds the risk of progression to kidney failure [1]. Epidemiological studies have shown that a decline in kidney function is associated with an increased risk of cardiovascular events and mortality. In addition, independent of kidney function, increased urinary albumin excretion has been associated with all-cause and cardiovascular mortality [4,5].

Large cohort studies over the past two decades have demonstrated increased risks of cardiovascular events, kidney disease progression, and mortality in patients with predialysis CKD [6,7,8]. In the Japanese CKD-JAC and Korean KNOW-CKD cohorts, advanced stages of CKD were associated with increased cardiovascular events and mortality [6,7]. Similarly, data from the CRIC cohort showed that the severity of albuminuria is associated with both renal and cardiovascular outcomes and that temporal variability in kidney function can independently predict the risk of cardiovascular disease, end-stage kidney failure, and mortality [8,9,10]. These findings underscore the close interplay between kidney and cardiovascular disease in CKD, which share several underlying pathophysiological mechanisms [11].

Although the kidney and cardiovascular risks associated with CKD are well established, long-term real-world studies evaluating major adverse kidney events (MAKE) and incident major adverse cardiovascular events (MACE) in parallel within the same predialysis CKD cohort remain limited. Such an approach may provide a more integrated view of the long-term cardiorenal burden and the potentially distinct factors associated with kidney and cardiovascular outcomes. Therefore, the present study aimed to characterize the long-term burden and clinical factors associated with MAKE and incident MACE in patients with predialysis CKD. As a secondary objective, we explored the associations between reno-cardioprotective therapies and these outcomes.

## 2. Materials and Methods

### 2.1. Study Design and Population

This retrospective observational cohort study aimed to evaluate long-term renal and cardiovascular outcomes in patients with predialysis CKD. Adult patients aged ≥18 years with stage 2–5 CKD who had been under regular follow-up in the nephrology outpatient clinic since January 2010 and had sufficient longitudinal medical records to allow at least 5 years of outcome assessment were eligible for inclusion. Patients receiving dialysis or with a history of kidney transplantation at baseline, as well as those with pre-existing coronary artery disease, heart failure, stroke, or malignancy, were excluded. Patients with insufficient longitudinal records, missing data required for outcome or covariate assessment, or inadequate follow-up to reliably ascertain study outcomes were not included in the analytical cohort. Follow-up data were retrospectively collected through January 2026.

The definition and classification of CKD were based on the 2024 Kidney Disease: Improving Global Outcomes (KDIGO) criteria. CKD was defined as an estimated glomerular filtration rate (eGFR) below 60 mL/min/1.73 m^2^ for at least three months and/or the presence of markers indicating structural or functional kidney abnormalities. Disease staging was performed according to the KDIGO classification: G5, eGFR < 15 mL/min/1.73 m^2^; G4, eGFR 15–29 mL/min/1.73 m^2^; G3b, eGFR 30–44 mL/min/1.73 m^2^; G3a, eGFR 45–59 mL/min/1.73 m ^2^; and G2, eGFR 60–89 mL/min/1.73 m^2^ [1].

The etiology of CKD was determined retrospectively based on information documented in the medical records prior to or at the time of CKD diagnosis. The etiological classification was based on the primary clinical diagnosis recorded by the attending nephrologist and was supported by the existing medical history, laboratory findings, imaging studies, and histopathological data, where available. Only those conditions identified as CKD etiologies that had arisen before or concurrently with the onset of CKD were considered, and reclassification based on clinical events occurring during follow-up was not performed.

Regular follow-up was defined as the availability of serial nephrology outpatient records sufficient to assess annual kidney function and ascertain MAKE, MACE, and censoring status during follow-up.

Cases lost to follow-up were defined as patients who did not attend follow-up visits during the observation period or who had insufficient follow-up data in their electronic medical records.

The study was reported with reference to the Strengthening the Reporting of Observational Studies in Epidemiology (STROBE) recommendations.

### 2.2. Data Collection

The demographic characteristics, comorbidities, chronic kidney disease etiology, baseline laboratory values, and initial treatments of the patients were obtained from the electronic medical records. Baseline kidney function was assessed using eGFR. Proteinuria and albuminuria were recorded using spot urine protein/creatinine and albumin/creatinine ratios, respectively. During follow-up, annual eGFR values, the final predialysis eGFR value, MAKE, MACE, death, and the dates of exit from predialysis follow-up were documented. In addition, the initiation dates for SGLT2 inhibitor and ACE inhibitor/ARB therapies started during follow-up were recorded.

eGFR was automatically calculated and reported by the hospital central biochemistry laboratory using the creatinine-based CKD-EPI equation in use during the study period and was extracted directly from the laboratory records. eGFR values were not retrospectively recalculated using a newer equation.

Baseline clinical and laboratory values were defined as those obtained at the first nephrology outpatient visit at cohort entry. Urine albumin-to-creatinine ratio (UACR) was recorded from the measurement obtained at this baseline visit and was not longitudinally analyzed during follow-up. Albuminuria was categorized as normal (<30 mg/g), microalbuminuria (30–300 mg/g), or macroalbuminuria (>300 mg/g) according to baseline UACR. When quantitative UACR was unavailable, albuminuria category was assigned using the baseline semiquantitative urine dipstick protein result. Dipstick protein +1 was categorized as microalbuminuria and ≥+2 as macroalbuminuria. This dipstick-based classification was used only as a surrogate for albuminuria category when quantitative UACR was unavailable and was not considered quantitatively equivalent to UACR measurement.

Longitudinal kidney function was assessed using eGFR values recorded at baseline and at years 1, 2, and 3, as well as the last available follow-up eGFR measurement. These longitudinal measurements were also used to estimate the individual annual eGFR slope.

Potential MACEs were identified from hospital records and diagnostic codes and were subsequently verified using the available clinical documentation, laboratory data, and imaging findings. Myocardial infarction and stroke diagnoses were accepted when supported by the corresponding clinical records and relevant laboratory and/or imaging findings. Cardiovascular death was classified according to the documented cause of death in the medical record.

### 2.3. Follow-Up Duration and Censoring

Follow-up time was calculated from the baseline assessment date to the date of the relevant study outcome or censoring. For patients without an event, censoring occurred at the earliest of the last documented outpatient visit, end of the study period, death, or exit from predialysis follow-up because of chronic dialysis or kidney transplantation, as applicable to the outcome being analyzed. For the primary MACE analysis, patients who initiated chronic dialysis or underwent kidney transplantation were censored at the date of exit from predialysis follow-up. Follow-up duration was summarized as median days, and incidence rates of MAKE and MACE were calculated as the number of events per 1000 person-years.

### 2.4. Outcomes

MAKE was defined as initiation of chronic dialysis, renal transplantation, or a ≥50% decline in baseline eGFR. The MAKE date was considered the date on which the first of these components occurred. The MAKE components were not evaluated as mutually exclusive categories.

MACE was defined as newly developed non-fatal myocardial infarction, non-fatal stroke, or cardiovascular death during the follow-up period. Since patients with a history of coronary artery disease or stroke at baseline were excluded, MACEs were evaluated as newly developed cardiovascular events that occurred during follow-up. The MACE components were not evaluated as mutually exclusive categories.

For descriptive analyses, component-specific event dates were additionally reviewed to identify the first qualifying MAKE and MACE component and to characterize overlap between individual components.

Cardiovascular death was considered a MACE component only when it occurred during the pre-dialysis follow-up period. Cardiovascular deaths that occurred after dialysis or renal transplantation were not included in the main MACE analysis; these patients were censored at the date of exit from the pre-dialysis follow-up.

### 2.5. Treatment Variables

SGLT2 inhibitor and ACE inhibitor/ARB use at cohort entry were recorded from the electronic medical records. For patients who initiated these treatments during follow-up, treatment initiation dates were determined from the electronic prescription and medical records. Treatment exposure was modeled as a time-dependent variable. Patients receiving the relevant treatment at cohort entry were considered exposed from time zero. Patients who initiated treatment during follow-up contributed unexposed person-time until the treatment initiation date and exposed person-time thereafter, whereas patients who never received the treatment remained unexposed throughout follow-up. For patients already receiving treatment at cohort entry, the exact duration of treatment before cohort entry could not be reliably determined; therefore, these patients were considered exposed from the date of cohort entry.

### 2.6. Statistical Analysis

IBM SPSS Statistics (version 27.0; IBM Corp., Armonk, NY, USA) was used for data analysis. GraphPad Prism (version 9; GraphPad Software, San Diego, CA, USA) was used for figure preparation and statistical analysis. Descriptive statistics for the data obtained were presented as mean ± standard deviation or median (interquartile range) for continuous variables and as numbers and percentages for categorical variables. The distributions of the times to development of MAKE and MACE according to clinical variables were evaluated using the Kaplan–Meier method, and comparisons between groups were performed using the log-rank (Mantel–Cox) test. Survival times in the Kaplan–Meier analysis were reported as median and 95% confidence intervals. In addition to analyses according to sex, diabetes mellitus, hypertension, CKD stage, and albuminuria category, prespecified subgroup analyses compared patients with diabetic kidney disease (DKD), defined as CKD attributed to diabetes mellitus, with those with non-DKD. Because only one patient had stage 5 CKD, this patient was combined with stage 4 for stage-specific survival analyses.

Multivariable Cox proportional hazards regression models were constructed separately for MAKE and MACE. For MAKE, Model 1 included age, sex, diabetes mellitus, baseline eGFR, albuminuria category, and baseline systolic blood pressure. Model 2A additionally included SGLT2 inhibitor use as a time-dependent covariate and calendar period of cohort entry (≥2018), whereas Model 2B additionally included ACE inhibitor/ARB use as a time-dependent covariate and calendar period of cohort entry (≥2018). For MACE, Model 1 included age, diabetes mellitus, baseline eGFR, log-transformed UACR [log(UACR + 1)], and baseline systolic blood pressure and was stratified by sex. Model 2A additionally included time-dependent SGLT2 inhibitor use and calendar period of cohort entry (≥2018), and Model 2B additionally included time-dependent ACE inhibitor/ARB use and calendar period of cohort entry (≥2018).

Treatment exposure was modeled as a time-dependent covariate according to the documented treatment initiation date. Patients receiving the respective treatment at cohort entry were considered exposed from time zero; patients initiating treatment during follow-up contributed unexposed person-time before treatment initiation and exposed person-time thereafter. Patients who never received the treatment remained unexposed throughout follow-up. Calendar period was included to account for potential temporal changes in treatment availability and clinical practice.

The proportional hazards assumption was assessed using scaled Schoenfeld residuals. For MAKE, the global proportional hazards assumption was satisfied in all final models. In the MACE analyses, sex violated the proportional hazards assumption; therefore, the final MACE models were stratified by sex, allowing separate baseline hazards while estimating common effects for the remaining covariates. After sex stratification, the global proportional hazards assumption was satisfied in all final MACE models. Multicollinearity was evaluated using variance inflation factors (VIFs). Model stability was additionally assessed by reporting the number of events, number of estimated parameters, and events per parameter. Model discrimination was assessed using Harrell’s concordance index (C-index). Model calibration was assessed at 5 years by comparing mean predicted event probabilities with Kaplan–Meier-observed event probabilities overall and across quintiles of predicted risk.

Missing covariate data were handled using complete-case analysis without imputation because missingness was low among variables included in the multivariable models. The MAKE models included 260 patients with 95 events, whereas the MACE models included 254 patients with 52 events. Missing-data frequencies for baseline variables are reported in Table 1.

To account for competing events, Fine–Gray subdistribution hazard regression was performed as a sensitivity analysis using the same baseline covariates as the corresponding primary Cox models. For MAKE, death from any cause occurring before MAKE was treated as a competing event. For MACE, chronic dialysis, kidney transplantation, and non-cardiovascular death occurring before MACE was treated as a competing event. Cause-specific hazard ratios and subdistribution hazard ratios (sHRs), with 95% confidence intervals and *p*-values, were reported.

Because SGLT2 inhibitor use was uncommon at cohort entry and was frequently initiated later during follow-up, an additional sensitivity analysis was performed among patients entering the cohort in 2018 or later. These models used the same covariates as Model 2A except for the calendar-period variable, which was omitted because all patients belonged to the same period. Owing to the limited number of outcome events in this restricted cohort, these analyses were considered exploratory.

Individual annual eGFR slopes were estimated using available longitudinal eGFR measurements obtained at baseline and at years 1, 2, and 3 of follow-up and were expressed as mL/min/1.73 m^2^ per year. eGFR slopes were compared between patients with and without MAKE and MACE using the Mann–Whitney U test. To evaluate the association between early eGFR trajectory and subsequent outcomes while preserving temporal ordering, a 3-year landmark analysis was additionally performed. Only patients who remained under observation at the 3-year landmark were included, and subsequent MAKEs and MACEs occurring after the landmark were analyzed using Cox proportional hazards models. Multivariable models were adjusted for age, sex, diabetes mellitus, baseline eGFR, albuminuria, and systolic blood pressure; the MACE model was stratified by sex. The proportional hazards assumption was assessed using Schoenfeld residuals.

A two-sided *p* value < 0.05 was considered statistically significant.

## 3. Results

A total of 263 predialysis CKD patients were included in this study. The mean age of the patients was 63.61 ± 12.60 years, with 134 female (51.0%) and 129 male (49.0%) included in the study. The baseline demographic, laboratory, and clinical characteristics of the patients are shown in Table 1.

The median follow-up duration was 2829 days. MAKE occurred in 97 patients (36.9%), corresponding to an incidence rate of 47.8 events per 1000 person-years. A ≥50% decline in eGFR occurred in all 97 patients with MAKE; 57 patients (21.7%) underwent permanent dialysis and 5 (1.9%) underwent kidney transplantation. Based on component-specific event dates, a ≥50% decline in eGFR was the first qualifying MAKE component in 95 patients (97.9%), permanent dialysis was the first component in one patient (1.0%), and dialysis and a ≥50% decline in eGFR occurred on the same date in one patient (1.0%). The overlap between MAKE components is shown in Table 2.

MACE occurred in 53 patients (20.2%), corresponding to an incidence rate of 26.0 events per 1000 person-years. Myocardial infarction occurred in 30 patients (11.4%), stroke in 16 (6.1%), and cardiovascular death in 12 (4.6%). Myocardial infarction was the first qualifying MACE component in 30 patients (56.6%), stroke in 14 (26.4%), and cardiovascular death in 9 (17.0%). Component overlap is detailed in Table 2.

**Figure 1:**
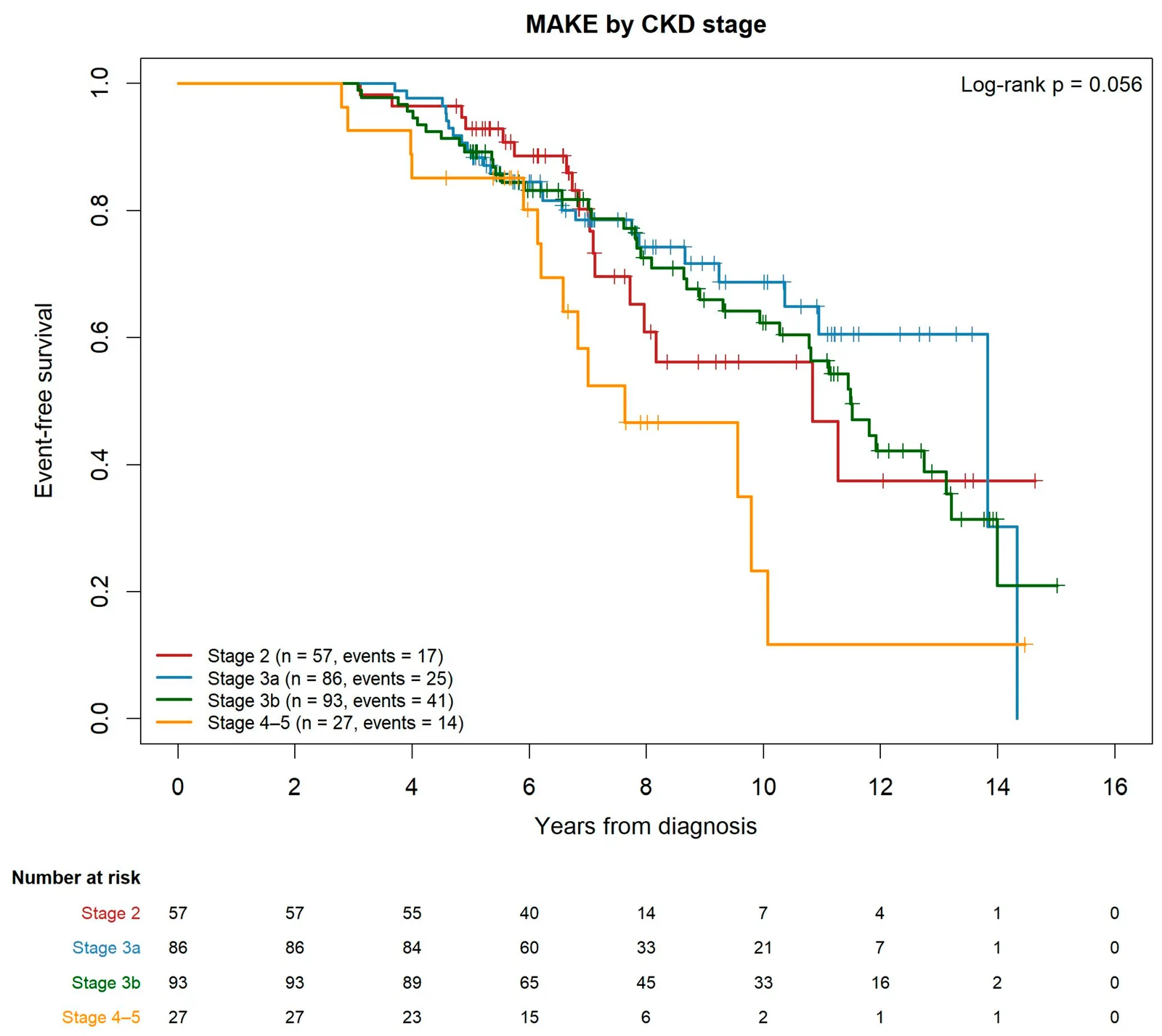
MAKE-free survival according to CKD stage (Log-rank *p* = 0.056). Figure legend: Stage 5 was combined with Stage 4 because only one patient had Stage 5 CKD. Numbers at risk are shown below Kaplan–Meier curve.

**Figure 2:**
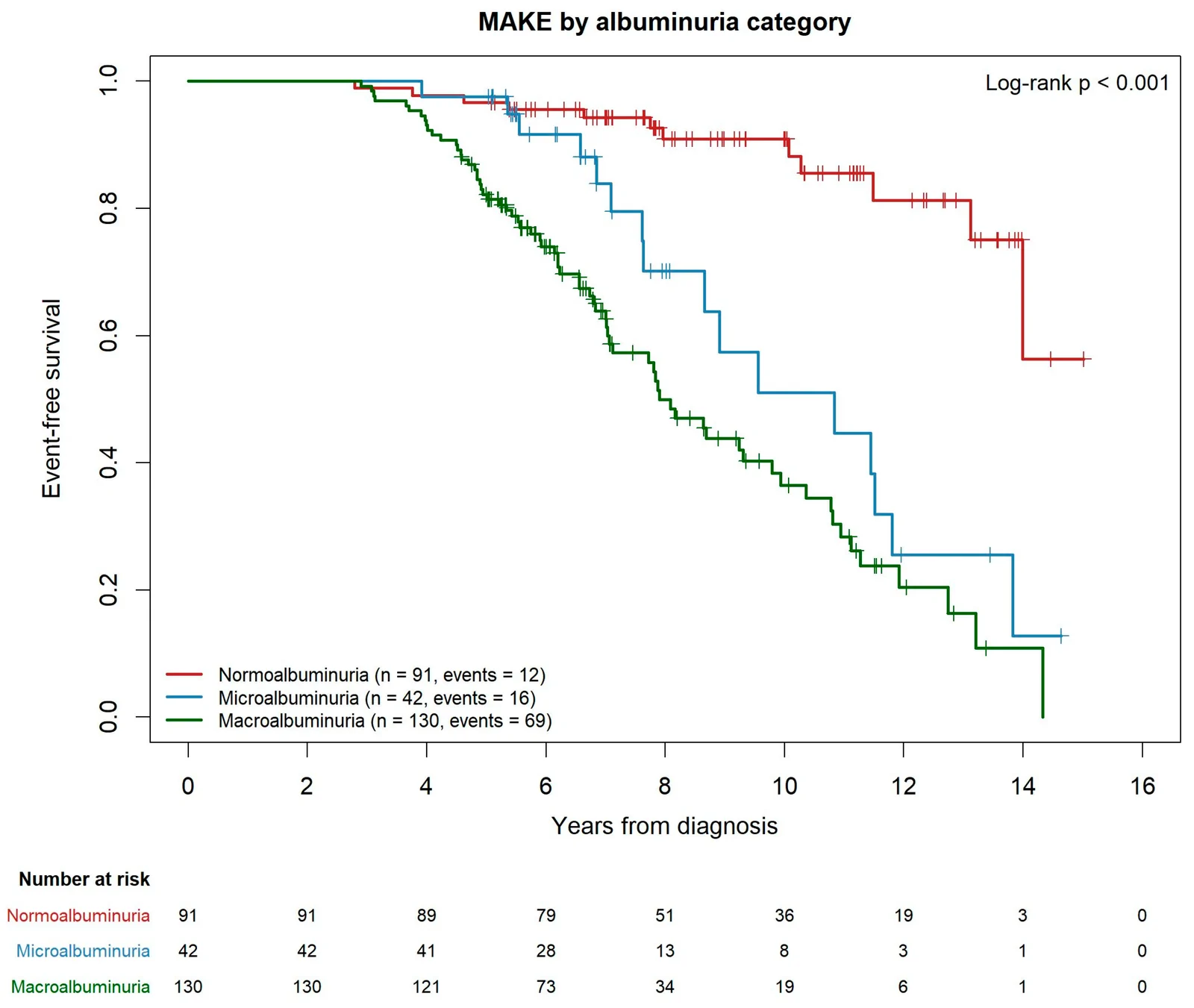
MAKE-free survival according to albuminuria category (Log-rank *p* < 0.001). Numbers at risk are shown below the Kaplan–Meier curve.

In the Kaplan–Meier analysis, the time to MAKE development was evaluated according to clinical variables (Table 3). MAKE rates tended to increase with advancing CKD stage, although the difference did not reach statistical significance (Log-rank *p* = 0.056) (Figure 1). Similarly, as the degree of albuminuria increased, a significant increase in the development of MAKE was detected (Log-rank *p* < 0.001) (Figure 2).

**Figure 3:**
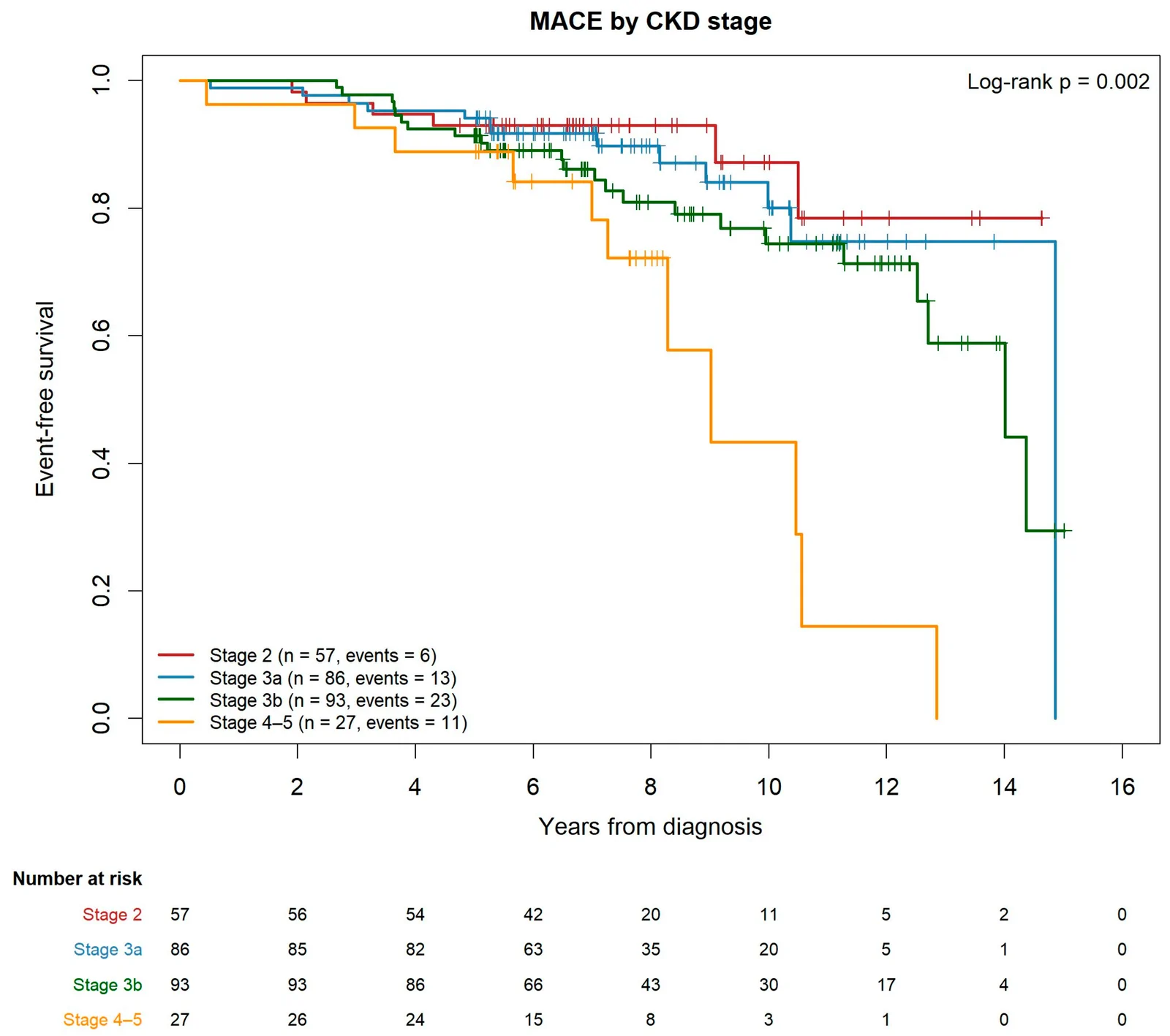
MACE-free survival according to CKD stage (Log-rank *p* = 0.002). Figure legend: Stage 5 was combined with Stage 4 because only one patient had Stage 5 CKD. Numbers at risk are shown below the Kaplan–Meier curve.

When the time to MACE development was evaluated according to clinical variables in Kaplan–Meier analysis (Table 3), DM was significantly associated with MACE development. MACE incidence was 29.2% in patients with DM and 14.0% in those without DM (log-rank *p* < 0.001). CKD stage was also significantly associated with MACE (log-rank *p* = 0.002), with MACE incidence increasing from 10.5% in stage 2 to 15.1% in stage 3a, 24.7% in stage 3b, and 40.7% in stage 4 (Figure 3).

In the multivariable Cox regression analyses for MAKE, lower baseline eGFR and greater albuminuria severity were independently associated with MAKE. In Model 1, each 10 mL/min/1.73 m^2^ higher baseline eGFR was associated with a lower hazard of MAKE (HR 0.84, 95% CI 0.72–0.98; *p* = 0.024). Compared with normal albuminuria, microalbuminuria was associated with approximately four-fold higher hazard (HR 4.15, 95% CI 1.91–9.00; *p* < 0.001), whereas macroalbuminuria was associated with nearly eight-fold higher hazard (HR 7.73, 95% CI 4.01–14.90; *p* < 0.001). These associations remained similar after additional adjustment for time-dependent SGLT2 inhibitor use or ACE inhibitor/ARB use and calendar period of cohort entry. Neither time-dependent SGLT2 inhibitor use (Model 2A: HR 0.59, 95% CI 0.31–1.11; *p* = 0.101) nor time-dependent ACE inhibitor/ARB use (Model 2B: HR 0.89, 95% CI 0.52–1.54; *p* = 0.682) was independently associated with MAKE (Table 4).

In the sex-stratified multivariable Cox analyses for MACE, older age and diabetes mellitus were consistently associated with higher MACE risk. In Model 1, each 10-year increase in age was associated with a 52% higher hazard of MACE (HR 1.52, 95% CI 1.12–2.06; *p* = 0.007), and diabetes mellitus was associated with approximately three-fold higher hazard (HR 3.00, 95% CI 1.60–5.62; *p* < 0.001). Baseline eGFR showed a borderline association in Model 1 (HR per 10 mL/min/1.73 m^2^ increase 0.77, 95% CI 0.60–1.00; *p* = 0.051), but was significantly associated with lower MACE risk in Models 2A and 2B (HR 0.76, *p* = 0.037 and *p* = 0.032, respectively). Neither log-transformed UACR nor systolic blood pressure was independently associated with MACE. Time-dependent SGLT2 inhibitor use (HR 1.28, 95% CI 0.55–2.94; *p* = 0.567) and time-dependent ACE inhibitor/ARB use (HR 1.33, 95% CI 0.57–3.13; *p* = 0.507) were also not significantly associated with MACE (Table 5).

Harrell’s C-index was 0.73 (95% CI, 0.68–0.78) for the MAKE Model 1 and 0.72 (95% CI, 0.63–0.80) for the sex-stratified MACE Model 1, indicating moderate discriminative ability. At 5 years, mean predicted and Kaplan–Meier-observed event risks were 10.0% and 10.4%, respectively, for MAKE and 6.6% and 7.5%, respectively, for MACE, indicating generally close agreement between predicted and observed risks. Calibration across risk quintiles was also broadly acceptable, although some deviation was observed in intermediate-risk groups, particularly for MACE.

Competing-risk sensitivity analyses using Fine–Gray regression yielded results that were broadly consistent with the primary Cox models. For MAKE, lower baseline eGFR remained associated with lower risk per 10 mL/min/1.73 m^2^ increase (sHR 0.84, 95% CI 0.72–0.98; *p* = 0.024), while microalbuminuria (sHR 4.29, 95% CI 2.09–8.82; *p* < 0.001) and macroalbuminuria (sHR 7.97, 95% CI 4.08–15.57; *p* < 0.001) remained strongly associated with higher MAKE risk. For MACE, older age (sHR 1.48, 95% CI 1.10–1.98; *p* = 0.009), diabetes mellitus (sHR 2.70, 95% CI 1.48–4.94; *p* = 0.001), and lower baseline eGFR (sHR 0.77, 95% CI 0.60–0.98; *p* = 0.033) remained significantly associated with the outcome. There were 47 competing events for MACE in the overall cohort (46 in the complete-case Fine–Gray model) and 3 competing events for MAKE (Table S1).

SGLT2 inhibitor exposure occurred in 75 patients (28.5%); only 9 patients (3.4%) were receiving treatment at cohort entry, whereas 66 (25.1%) initiated treatment during follow-up. Among new users, the median time from cohort entry to treatment initiation was 5.0 years (IQR, 3.3–7.4), and the median exposure duration was 3.1 years (IQR, 1.6–4.0) (Table S2A).

In the sensitivity analysis restricted to patients entering the cohort in 2018 or later, SGLT2 inhibitor use remained unassociated with either MACE (HR 1.14, 95% CI 0.26–4.90; *p* = 0.861) or MAKE (HR 1.04, 95% CI 0.35–3.07; *p* = 0.940). However, the low events-per-parameter ratios in these restricted analyses (1.9 for MACE and 2.8 for MAKE) limited the precision and stability of these estimates (Table S2B).

The median annual eGFR slope in the overall cohort was −2.0 mL/min/1.73 m^2^/year (IQR, −4.6 to −0.3). Patients who developed MAKE had a significantly faster eGFR decline than those who did not [−3.7 (IQR, −5.8 to −1.8) vs. −1.2 (IQR, −3.4 to 0.2) mL/min/1.73 m^2^/year; *p* < 0.001]. In contrast, patients who developed MACE had a less pronounced eGFR decline than those without MACE [−0.6 (IQR, −3.2 to 0.7) vs. −2.6 (IQR, −4.7 to −0.6) mL/min/1.73 m^2^/year; *p* = 0.003] (Table S3A).

In the 3-year landmark analysis, faster eGFR decline was independently associated with subsequent MAKE (adjusted HR per 1 mL/min/1.73 m^2^/year faster decline, 1.29; 95% CI, 1.20–1.39; *p* < 0.001), whereas no significant association was observed with subsequent MACE (adjusted HR, 0.94; 95% CI, 0.84–1.05; *p* = 0.243) (Table S3B). The global proportional hazards tests were non-significant for both the MACE (*p* = 0.128) and MAKE (*p* = 0.051) models.

Additional adjustment for baseline HbA1c did not materially alter the associations in the MACE model, and HbA1c itself was not independently associated with MACE (Table S4).

## 4. Discussion

In this long-term real-world cohort of patients with predialysis CKD, MAKE and MACE were both frequent, but their associated clinical profiles differed. Greater albuminuria severity and lower baseline eGFR were independently associated with MAKE, whereas older age, diabetes mellitus, and lower baseline eGFR were associated with incident MACE. These findings suggest that renal and cardiovascular risk in predialysis CKD is partly overlapping but not identical, highlighting the importance of evaluating kidney and cardiovascular outcomes in parallel. Treatment-related findings were less conclusive and should be interpreted cautiously because of the observational design and limited treatment exposure.

The incidence rates of MAKE and MACE were 47.8 and 26.0 per 1000 person-years, respectively, underscoring the substantial long-term cardiorenal burden in predialysis CKD. The CKD-JAC study reported increasing cardiovascular risk with declining kidney function, particularly in advanced CKD and among patients with diabetes [6]. Consistent with these findings, MACE rates in our cohort increased from 10.5% in stage 2 to 40.7% in stage 4 CKD. However, when baseline kidney function was modeled continuously in the multivariable analysis, lower baseline eGFR was independently associated with MACE. This finding supports the prognostic value of baseline kidney function as a continuous marker of cardiovascular risk.

Diabetes mellitus was another strong independent factor associated with MACE. Patients with diabetes had approximately a three-fold higher hazard of MACE after multivariable adjustment. This finding is consistent with observations from CKD-JAC, KNOW-CKD, and CRIC, in which diabetes was associated with a greater cardiovascular event burden among patients with CKD [6,7,8].

In subgroup analyses according to CKD etiology, MAKE-free survival did not differ significantly between patients with DKD and non-DKD, whereas MACE was more frequent among patients with DKD. This finding is consistent with the greater cardiovascular risk associated with diabetes in CKD and supports the independent association between diabetes and MACE observed in our multivariable models. The median annual eGFR decline in the overall cohort was −2.0 mL/min/1.73 m^2^/year. Although patients who developed MACE showed a less pronounced eGFR decline than those without MACE in the descriptive analysis, eGFR slope was not independently associated with subsequent MACE in the 3-year landmark analysis. This finding suggests that longitudinal eGFR decline did not provide additional prognostic information for subsequent cardiovascular events in this cohort.

Older age was also independently associated with MACE, consistent with previous studies demonstrating an age-related increase in cardiovascular risk in both non-dialysis CKD populations and broader population-based cohorts [12,13].

In the CRIC cohort, cardiovascular and kidney event rates were higher than those observed in our study, and both outcomes were more frequent among patients with diabetes [8]. Differences in event rates may reflect variations in baseline risk, CKD severity, comorbidity burden, and treatment patterns. Similarly, the KNOW-CKD study reported increasing cardiovascular event rates with advancing CKD stage, particularly among patients with diabetes [7]. Taken together, these findings are broadly consistent with the cardiovascular risk pattern observed in our cohort.

A recent analysis from the Fukuoka Kidney Disease Registry showed that greater multimorbidity was associated with higher risks of MACE and all-cause mortality in CKD [14]. Although multimorbidity was not specifically assessed in our cohort, the independent association between diabetes and MACE further supports the contribution of comorbid disease burden to cardiovascular risk in predialysis CKD.

Albuminuria was the strongest factor associated with MAKE in the present study and showed a clear graded association with kidney risk. Compared with normal albuminuria, microalbuminuria was associated with an approximately four-fold higher hazard of MAKE, whereas macroalbuminuria was associated with an approximately eight-fold higher hazard. This graded association is consistent with previous CKD studies demonstrating the prognostic importance of urinary albumin excretion for kidney disease progression [9,15,16]. Albuminuria may therefore provide clinically relevant prognostic information beyond conventional CKD staging for long-term kidney outcomes.

In contrast, albuminuria was not significantly associated with incident MACE in the Kaplan–Meier analysis in our cohort. Although albuminuria is well established as a cardiovascular risk marker in broader CKD populations [4,5], the absence of a significant association in the present study may partly reflect the limited number of cardiovascular events and the exclusion of patients with established coronary artery disease or previous stroke. Therefore, this finding should not be interpreted as evidence that albuminuria lacks cardiovascular prognostic relevance.

Lower baseline eGFR was independently associated with MAKE in the multivariable analysis; each 10 mL/min/1.73 m^2^ increase in baseline eGFR was associated with a lower hazard of MAKE. This finding underscores the prognostic importance of baseline kidney function for long-term renal outcomes in predialysis CKD. Importantly, the 3-year landmark analysis further showed that faster eGFR decline was independently associated with subsequent MAKE, suggesting that longitudinal kidney function trajectory may provide prognostic information beyond a single baseline measurement. Consistent with this observation, previous real-world CKD cohorts have reported higher risks of ESKD or death among patients with poorer kidney function and other adverse clinical characteristics, including diabetes, hypertension, and male sex [17].

In the time-dependent Cox analyses, neither SGLT2 inhibitor nor ACE inhibitor/ARB use was independently associated with MAKE or MACE. These findings should be interpreted cautiously given the retrospective observational design of the study. Randomized trials, including CREDENCE, DAPA-CKD, and EMPA-KIDNEY, have demonstrated substantial renal and cardiovascular benefits of SGLT2 inhibitors in patients with CKD [18,19,20,21,22,23,24]. Therefore, the absence of statistically significant associations in our cohort should not be interpreted as evidence of lack of clinical benefit. Our ability to evaluate outcomes associated with SGLT2 inhibitor use was limited by the exposure pattern. Only nine patients were receiving an SGLT2 inhibitor at cohort entry, whereas most exposed patients initiated treatment later during follow-up, at a median of 5.0 years after cohort entry. The median exposure duration was 3.1 years, and relatively few MAKEs and MACEs occurred during exposed person-time, resulting in limited statistical power and wide confidence intervals. In addition, treatment initiation was not randomized and may have been influenced by kidney function, diabetes status, cardiovascular risk, and evolving prescribing practices, raising the possibility of confounding by indication. Detailed information regarding dose changes, adherence, temporary discontinuation, restart, and treatment persistence was also not consistently available. Similar limitations apply to ACE inhibitor/ARB exposure, particularly given the high prevalence of use and the inability of retrospective data to fully characterize treatment intensity and persistence. Accordingly, the treatment-related findings in this study should be regarded as observational associations rather than causal estimates of treatment effect.

Because the cohort included patients followed since 2010, changes in CKD management and treatment availability over time represented a potential source of calendar-time confounding. The year 2018 was used as a pragmatic calendar-period cut-off, reflecting changes in national reimbursement regulations and prescribing patterns for SGLT2 inhibitors in Türkiye. We therefore additionally adjusted the multivariable models for the calendar period of cohort entry. Calendar period was not independently associated with either MAKE or MACE in the revised models. Nevertheless, adjustment for a broad calendar period cannot fully account for secular changes in clinical practice, prescribing patterns, or treatment indications. Residual confounding related to treatment era may therefore remain.

The cardiovascular event rate observed in this cohort should also be interpreted in the context of the study selection criteria. Patients with pre-existing coronary artery disease, heart failure, or stroke were excluded to specifically evaluate incident MACE rather than recurrent cardiovascular events. However, this approach selected a population with a lower baseline cardiovascular risk than an unselected predialysis CKD population. Consequently, the observed MACE incidence may underestimate the overall cardiovascular burden of predialysis CKD and may not be directly generalizable to patients with established cardiovascular disease. The exclusion of patients with malignancy may have further reduced the overall comorbidity burden of the study population.

This study has several limitations. First, the retrospective, single-center design limits causal inference and leaves the possibility of residual and unmeasured confounding. Although SGLT2 inhibitor and ACE inhibitor/ARB exposure was modeled as time-dependent according to treatment initiation dates, detailed information on dose, adherence, treatment interruptions, restart, and persistence was not consistently available. Furthermore, treatment duration before cohort entry could not be reliably determined for patients already receiving therapy at baseline.

Second, the long study period was accompanied by changes in CKD treatment practices and medication availability. Although calendar period of cohort entry was included in the multivariable models, this adjustment may not fully account for secular changes in clinical practice or confounding by indication. Third, the relatively small number of MACEs limited statistical power and constrained the complexity of multivariable cardiovascular modeling. Because sex violated the proportional hazards assumption, the final MACE models were stratified by sex; nevertheless, the limited number of events, particularly in treatment-focused and restricted sensitivity analyses, reduced the precision of effect estimates.

Fourth, although baseline HbA1c was available, it was not included in the final MACE model in order to preserve model parsimony and reduce the risk of overfitting. Longitudinal information on glycemic control, albuminuria, and blood pressure was incomplete, while smoking history and baseline lipid levels were not consistently available and therefore could not be incorporated into the multivariable analyses. In a small proportion of patients, albuminuria classification was based on semiquantitative dipstick protein rather than quantitative UACR, which may have introduced some degree of exposure misclassification. Residual confounding related to these cardiovascular risk factors therefore cannot be excluded.

Patients with pre-existing heart failure were excluded at baseline; therefore, prior heart failure was not evaluated as a covariate. CKD etiology was based predominantly on clinical records rather than histopathological confirmation. Finally, exclusion of patients with pre-existing coronary artery disease, heart failure, stroke, or malignancy resulted in a selected population with a lower baseline cardiovascular and comorbidity burden. Consequently, the observed MACE incidence may underestimate the overall cardiovascular burden of an unselected predialysis CKD population, and the generalizability of these findings should be interpreted accordingly.

Despite these limitations, this study provides long-term real-world data on kidney and cardiovascular outcomes within the same predialysis CKD cohort. The strong graded association between albuminuria and MAKE and the associations of age, diabetes, and baseline kidney function with incident MACE highlight the partly distinct clinical profiles underlying renal and cardiovascular risk in CKD.

## 5. Conclusions

In conclusion, this long-term real-world observational study jointly evaluated kidney disease progression and incident cardiovascular events in patients with predialysis CKD. Greater albuminuria severity was strongly associated with MAKE, whereas older age, diabetes mellitus, and lower baseline eGFR were independently associated with MACE. After accounting for treatment timing using time-dependent Cox regression, neither SGLT2 inhibitor nor ACE inhibitor/ARB use was independently associated with MAKE or MACE. These findings suggest that renal and cardiovascular outcomes in predialysis CKD may be associated with partly distinct clinical risk profiles. Given the retrospective, single-center design, limited treatment exposure, and relatively small number of cardiovascular events, the treatment-related findings should be interpreted cautiously and require confirmation in larger prospective studies.

## Figures and Tables

**Table 1:** Baseline demographic, laboratory, and clinical characteristics of the patients.

Variable	Values	Missing, *n* (%)
Age, years, mean ± SD	63.61 ± 12.60	0 (0.0)
Sex, *n* (%) female/male	134 (51.0)/129 (49.0)	0 (0.0)
Body mass index, kg/m^2^, median (IQR)	29.37 (26.61–32.77)	132 (50.2)
Diabetes mellitus, *n* (%)	106 (40.3)	0 (0.0)
Hypertension, *n* (%)	230 (87.5)	0 (0.0)
Systolic blood pressure, mmHg, median (IQR)	136.50 (120.00–151.00)	3 (1.1)
Diastolic blood pressure, mmHg, median (IQR)	80.00 (71.00–90.00)	3 (1.1)
Baseline eGFR, mL/min/1.73 m^2^, median (IQR)	46.00 (35.50–58.00)	0 (0.0)
Urine albumin-to-creatinine ratio, mg/g, median (IQR)	279.00 (20.00–788.00)	6 (2.3)
Urine protein-to-creatinine ratio, mg/g, median (IQR)	710.00 (200.00–1830.00)	2 (0.8)
Hemoglobin, g/dL, mean ± SD	12.94 ± 1.84	0 (0.0)
Albumin, g/dL, median (IQR)	4.20 (3.90–4.40)	14 (5.3)
Creatinine, mg/dL, median (IQR)	1.45 (1.25–1.80)	0 (0.0)
Sodium, mmol/L, median (IQR)	139.00 (138.00–141.00)	0 (0.0)
Potassium, mmol/L, median (IQR)	4.70 (4.40–5.00)	0 (0.0)
Calcium, mg/dL, median (IQR)	9.40 (9.05–9.80)	0 (0.0)
Phosphorus, mg/dL, median (IQR)	3.30 (3.00–3.80)	0 (0.0)
Parathyroid hormone, pg/mL, median (IQR)	70.00 (45.25–105.75)	5 (1.9)
HbA1c, %, median (IQR)	5.80 (5.00–6.80)	0 (0.0)
CKD etiology, *n* (%)		0 (0.0)
Diabetes mellitus	89 (33.8)	—
Hypertension	76 (28.9)	—
Polycystic kidney disease	14 (5.3)	—
Glomerulonephritis	59 (22.4)	—
Other	25 (9.5)	—
CKD stage, *n* (%)		0 (0.0)
Stage 2	57 (21.7)	—
Stage 3a	86 (32.7)	—
Stage 3b	93 (35.4)	—
Stage 4	26 (9.9)	—
Stage 5	1 (0.4)	—

Note: Percentages of missing data were calculated using the total cohort (*n* = 263) as the denominator. Abbreviations: CKD, chronic kidney disease; eGFR, estimated glomerular filtration rate; HbA1c, glycated hemoglobin; SD, standard deviation; IQR, interquartile range.

**Table 2:** MAKE and MACE outcomes during follow-up and incidence rates per 1000 person-years.

Variable	Value
Follow-up duration, median (IQR), days	2829 (2252.5–3916)
MAKE	
Overall MAKE, *n* (%)	97 (36.9)
≥50% decline in eGFR, *n* (%)	97 (36.9)
Permanent dialysis, *n* (%)	57 (21.7)
Kidney transplantation, *n* (%)	5 (1.9)
First qualifying MAKE component, *n* (%)	
≥50% decline in eGFR	95 (97.9)
Permanent dialysis	1 (1.0)
≥50% eGFR decline and dialysis on the same date	1 (1.0)
Kidney transplantation	0 (0.0)
Overlap of MAKE components, *n*	
≥50% eGFR decline only	36
≥50% eGFR decline + permanent dialysis	56
≥50% eGFR decline + kidney transplantation	4
≥50% eGFR decline + dialysis + transplantation	1
Time to MAKE, median (IQR), days	2478 (1787–3373)
Total MAKE risk time, person-years	2028.1
Incidence, per 1000 person-years	47.8
MACE	
Overall MACE, *n* (%)	53 (20.2)
Myocardial infarction, *n* (%)	30 (11.4)
Stroke, *n* (%)	16 (6.1)
Cardiovascular death, *n* (%)	12 (4.6)
First qualifying MACE component, *n* (%)	
Myocardial infarction	30 (56.6)
Stroke	14 (26.4)
Cardiovascular death	9 (17.0)
Overlap of MACE components, *n*	
Myocardial infarction only	25
Stroke only	14
Cardiovascular death only	9
Myocardial infarction + stroke	2
Myocardial infarction + cardiovascular death	3
Time to MACE, median (IQR), days	2378 (1333–3353)
Total MACE risk time, person-years	2034.6
Incidence, per 1000 person-years	26.0

Note: MAKE and MACE components are not mutually exclusive; more than one component may have occurred in the same patient. Abbreviations: eGFR, estimated glomerular filtration rate; IQR, interquartile range; MACE, major adverse cardiovascular event; MAKE, major adverse kidney event.

**Table 3:** Kaplan–Meier Analysis of Time to MAKE and MACE According to Clinical Variables.

Variable	Group	Event, *n* (%)	Median (95% CI), Days	*p* Value
A. MAKE				
Sex	Female	50 (37.3)	4180 (3788–4572)	0.908
	Male	47 (36.4)	3996 (3113–4879)	
DM	No	57 (36.3)	4116 (3395–4837)	0.518
	Yes	40 (37.7)	3996 (3400–4592)	
HT	No	10 (30.3)	4116 (2454–5778)	0.951
	Yes	87 (37.8)	4060 (3657–4463)	
	Yes	15 (20.8)	4824 (NR)	
CKD etiology	Non-DKD	63 (36.2)	4116 (3679–5109)	0.591
	DKD	34 (38.2)	4193 (3171–5051)	
CKD stage	Stage 2	17 (29.8)	3956 (2579–5333)	0.056
	Stage 3a	25 (29.1)	5051 (3565–6537)	
	Stage 3b	41 (44.1)	4193 (3785–4601)	
	Stage 4–5	14 (51.9)	2787 (1814–3760)	
Albuminuria	Normal	12 (13.2)	NR	<0.001
	Microalbuminuria	16 (38.1)	3956.0 (2685.1–5226.9)	
	Macroalbuminuria	69 (53.1)	2887.0 (2396.9–3377.1)	
B. MACE				
Sex	Female	24 (17.9)	5118 (4579–5657)	0.379
	Male	29 (22.5)	5428 (3222–7634)	
DM	No	22 (14.0)	5118 (4537–5699)	<0.001
	Yes	31 (29.2)	NR	
HT	No	3 (9.1)	NR	0.175
	Yes	50 (21.7)	5118 (4456–5780)	
CKD etiology	Non-DKD	24 (13.8)	5249 (4640–NR)	<0.001
	DKD	29 (32.6)	3832 (3353–5428)	
CKD stage	Stage 2	6 (10.5)	NR	0.002
	Stage 3a	13 (15.1)	5428 (NR)	
	Stage 3b	23 (24.7)	5118 (4186–6050)	
	Stage 4–5	11 (40.7)	3293 (2671–3915)	
Albuminuria	Normal	19 (20.9)	5249.0 (4370.2–6127.8)	0.686
	Microalbuminuria	7 (16.7)	NR	
	Macroalbuminuria	27 (20.8)	5118.0 (4353.2–5882.8)	

Note: Comparisons were performed using the log-rank test. DKD was defined as CKD attributed to diabetes mellitus; all other CKD etiologies were classified as non-DKD. Because only one patient had stage 5 CKD, stage 5 was combined with stage 4 for stage-specific survival analyses. Abbreviations: CI, confidence interval; CKD, chronic kidney disease; DKD, diabetic kidney disease; DM, diabetes mellitus; HT, hypertension; MACE, major adverse cardiovascular event; MAKE, major adverse kidney event; NR, not reached.

**Table 4:** Multivariable Cox regression models for major adverse kidney events (MAKEs).

Variable	Model 1 HR (95% CI)	*p* Value	Model 2A HR (95% CI)	*p* Value	Model 2B HR (95% CI)	*p* Value
Age, per 10 years	0.94 (0.77–1.16)	0.559	0.94 (0.76–1.15)	0.522	0.94 (0.77–1.16)	0.585
Male sex	0.94 (0.61–1.44)	0.780	0.95 (0.62–1.46)	0.826	0.93 (0.61–1.44)	0.760
Diabetes mellitus	1.06 (0.69–1.64)	0.790	1.23 (0.78–1.94)	0.379	1.06 (0.69–1.64)	0.793
Baseline eGFR, per 10 mL/min/1.73 m^2^	0.84 (0.72–0.98)	0.024	0.83 (0.71–0.97)	0.019	0.84 (0.72–0.98)	0.028
Microalbuminuria (vs. normal)	4.15 (1.91–9.00)	<0.001	4.03 (1.86–8.75)	<0.001	4.09 (1.88–8.89)	<0.001
Macroalbuminuria (vs. normal)	7.73 (4.01–14.90)	<0.001	8.21 (4.25–15.84)	<0.001	7.77 (4.04–14.98)	<0.001
Systolic blood pressure, per 10 mmHg	1.04 (0.94–1.14)	0.482	1.03 (0.94–1.14)	0.517	1.03 (0.94–1.14)	0.531
SGLT2 inhibitor use, time-dependent	—	—	0.59 (0.31–1.11)	0.101	—	—
ACE inhibitor/ARB use, time-dependent	—	—	—	—	0.89 (0.52–1.54)	0.682
Cohort entry ≥2018	—	—	1.32 (0.74–2.36)	0.346	1.16 (0.66–2.03)	0.610

Notes: Model 1 included age, sex, diabetes mellitus, baseline eGFR, albuminuria category, and baseline systolic blood pressure. Model 2A additionally included time-dependent SGLT2 inhibitor use and calendar period of cohort entry (≥2018). Model 2B additionally included time-dependent ACE inhibitor/ARB use and calendar period of cohort entry (≥2018). Treatment exposure was modeled as a time-dependent covariate. All models were based on complete-case analysis (*n* = 260; 95 MAKEs). The global proportional hazards assumption was satisfied for Model 1 (*p* = 0.324), Model 2A (*p* = 0.565), and Model 2B (*p* = 0.428). Variance inflation factors were low in all models (maximum VIF = 1.34), indicating no evidence of relevant multicollinearity. The number of estimated parameters and events per parameter were 7 and 13.6 for Model 1, and 9 and 10.6 for Models 2A and 2B, respectively. Abbreviations: ACE, angiotensin-converting enzyme; ARB, angiotensin receptor blocker; CI, confidence interval; eGFR, estimated glomerular filtration rate; HR, hazard ratio; MAKE, major adverse kidney event; SGLT2, sodium-glucose cotransporter 2.

**Table 5:** Multivariable Cox regression models for major adverse cardiovascular events (MACEs).

Variable	Model 1 HR (95% CI)	*p* Value	Model 2A HR (95% CI)	*p* Value	Model 2B HR (95% CI)	*p* Value
Age, per 10 years	1.52 (1.12–2.06)	0.007	1.55 (1.14–2.10)	0.005	1.52 (1.12–2.06)	0.007
Male sex	Stratified	—	Stratified	—	Stratified	—
Diabetes mellitus	3.00 (1.60–5.62)	<0.001	2.85 (1.49–5.45)	0.002	2.92 (1.56–5.46)	<0.001
Baseline eGFR, per 10 mL/min/1.73 m^2^	0.77 (0.60–1.00)	0.051	0.76 (0.59–0.98)	0.037	0.76 (0.58–0.98)	0.032
log(UACR + 1)	1.07 (0.91–1.26)	0.420	1.05 (0.90–1.24)	0.531	1.06 (0.90–1.24)	0.493
Systolic blood pressure, per 10 mmHg	0.97 (0.84–1.11)	0.652	0.98 (0.85–1.13)	0.770	0.98 (0.85–1.13)	0.794
SGLT2 inhibitor use, time-dependent	—	—	1.28 (0.55–2.94)	0.567	—	—
ACE inhibitor/ARB use, time-dependent	—	—	—	—	1.33 (0.57–3.13)	0.507
Cohort entry ≥2018	—	—	1.51 (0.69–3.28)	0.298	1.55 (0.73–3.29)	0.258

Notes: Model 1 included age, diabetes mellitus, baseline eGFR, log-transformed UACR, and baseline systolic blood pressure and was stratified by sex. Model 2A additionally included time-dependent SGLT2 inhibitor use and calendar period of cohort entry (≥2018). Model 2B additionally included time-dependent ACE inhibitor/ARB use and calendar period of cohort entry (≥2018). Sex was used as a stratification variable because it violated the proportional hazards assumption and therefore no hazard ratio was estimated for sex. Treatment exposure was modeled as a time-dependent covariate. All models were based on complete-case analysis (*n* = 254; 52 MACEs). After stratification by sex, the global proportional hazards assumption was satisfied in Model 1 (*p* = 0.074), Model 2A (*p* = 0.183), and Model 2B (*p* = 0.229). Variance inflation factors were low in all models (maximum VIF = 1.31), indicating no evidence of relevant multicollinearity. The number of estimated parameters and events per parameter were 5 and 10.4 for Model 1, and 7 and 7.4 for Models 2A and 2B, respectively. Abbreviations:ACE, angiotensin-converting enzyme; ARB, angiotensin receptor blocker; CI, confidence interval; eGFR, estimated glomerular filtration rate; HR, hazard ratio; MACE, Major Adverse Cardiovascular Event; SGLT2, sodium-glucose cotransporter 2; UACR, Urine albumin-to-creatinine ratio.

## Data Availability

The datasets created and/or analyzed during the current study are not publicly available because they contain patient information, but data supporting the findings of this study can be obtained from the corresponding author (A.K.A.) upon reasonable request.

## Supplementary Materials

The following supporting information can be downloaded at: https://www.mdpi.com/article/10.3390/medicina62101957/s1, Table S1. Competing-Risk Sensitivity Analyses for MACE and MAKE; Table S2A. Exposure Characteristics for SGLT2 Inhibitors and ACE Inhibitors/ARBs; Table S2B. Sensitivity Analysis Restricted to Patients Entering the Cohort in 2018 or Later; Table S3A. eGFR Slope According to MAKE and MACE Status; Table S3B. Association of eGFR slope with subsequent outcomes in the 3-year landmark analysis, Table S4. Sensitivity Analysis of the Multivariable MACE Model Including Baseline HbA1c.

## Abbreviations

The following abbreviations are used in this manuscript:

ACE	Angiotensin-converting enzyme
ARB	Angiotensin receptor blocker
CI	Confidence interval
CKD	Chronic kidney disease
DM	Diabetes mellitus
eGFR	Estimated glomerular filtration rate
HR	Hazard ratio
HT	Hypertension
IQR	Interquartile range
KDIGO	Kidney Disease: Improving Global Outcomes
MACE	Major adverse cardiovascular events
MAKE	Major adverse kidney events
NR	Not reached
SD	Standard deviation
SGLT2	Sodium-glucose cotransporter 2
sHR	Subdistribution hazard ratio
