# Supplementary material for: Long-Term Major Adverse Kidney and Cardiovascular Events in Predialysis Chronic Kidney Disease: A Real-World Cohort Study
Source: Medicina (Kaunas). 2026 Oct 10;62(10):1957. doi: 10.3390/medicina62101957 (PMC13706874; doi:10.3390/medicina62101957)
Supplement: Supplementary file 1 [file medicina-62-01957-s001.zip › medicina-4571360-supplementary.pdf]

Supplementary Table S1. Competing-Risk Sensitivity Analyses for MACE and MAKE

Outcome / Variable	Cause-specific HR (95% CI)	p value	Subdistribution HR (95% CI)	p value
**MACE**				
Age, per 10 years	1.43 (1.06–1.91)	0.017	1.48 (1.10–1.98)	0.009
Male sex	1.42 (0.78–2.60)	0.250	1.28 (0.71–2.30)	0.409
Diabetes mellitus	2.92 (1.58–5.40)	<0.001	2.70 (1.48–4.94)	0.001
Baseline eGFR, per 10 mL/min/1.73 m^2	0.73 (0.57–0.93)	0.012	0.77 (0.60–0.98)	0.033
log(UACR + 1)	1.04 (0.89–1.22)	0.619	1.01 (0.86–1.19)	0.867
Systolic blood pressure, per 10 mmHg	0.95 (0.83–1.09)	0.471	0.95 (0.83–1.08)	0.423
**MAKE**				
Age, per 10 years	0.94 (0.77–1.16)	0.559	0.94 (0.77–1.14)	0.507
Male sex	0.94 (0.61–1.44)	0.780	0.95 (0.62–1.44)	0.801
Diabetes mellitus	1.06 (0.69–1.64)	0.790	1.06 (0.69–1.62)	0.794
Baseline eGFR, per 10 mL/min/1.73 m^2	0.84 (0.72–0.98)	0.024	0.84 (0.72–0.98)	0.024
Microalbuminuria (vs. normal)	4.15 (1.91–9.00)	<0.001	4.29 (2.09–8.82)	<0.001
Macroalbuminuria (vs. normal)	7.73 (4.01–14.90)	<0.001	7.97 (4.08–15.57)	<0.001
Systolic blood pressure, per 10 mmHg	1.04 (0.94–1.14)	0.482	1.03 (0.93–1.15)	0.517

Notes: *Cause-specific hazard ratios were estimated using Cox regression, whereas subdistribution hazard ratios were estimated using Fine–Gray competing-risk regression. For MACE, chronic dialysis, kidney transplantation, and non-cardiovascular death occurring before MACE were treated as competing events. There were 47 unique competing events in the overall cohort; 46 were included in the complete-case Fine–Gray model. For MAKE, death from any cause occurring before MAKE was treated as a competing event, with 3 competing events identified. The Fine–Gray analyses were performed as sensitivity analyses using the same baseline covariates as the corresponding primary Cox models.*

Abbreviations: *ACE, angiotensin-converting enzyme; ARB, angiotensin receptor blocker; CI, confidence interval; CKD, chronic kidney disease; DM, diabetes mellitus; HR, hazard ratio; MACE, major adverse cardiovascular events; MAKE, major adverse kidney events; SGLT2, sodium-glucose cotransporter 2; UACR, Urine albumin-to-creatinine ratio.*

Supplementary Table S2A. Exposure Characteristics for SGLT2 Inhibitors and ACE Inhibitors/ARBs

Exposure characteristic	SGLT2 inhibitor	ACE inhibitor/ARB
Patients exposed during follow-up, n (%)	75 (28.5)	229 (87.1)
At baseline, n (%)	9 (3.4)	180 (68.4)
Initiated during follow-up, n (%)	66 (25.1)	49 (18.6)
Calendar years of initiation among new users	2018 or later	2010 or later
Time from cohort entry to initiation, median (IQR), years	5.0 (3.3–7.4)	1.8 (0.4–3.7)
Duration of exposure, median (IQR), years	3.1 (1.6–4.0)	7.0 (5.5–10.4)
Total exposed person-years	242.5	1,822.0
MACE events during exposed person-time, n	9	45
MAKE events during exposed person-time, n	15	80

Note: *Treatment exposure was defined from cohort entry for patients receiving the respective therapy at baseline and from the documented treatment initiation date for new users during follow-up. For the combined ACE inhibitor/ARB category, patients receiving either an ACE inhibitor or an ARB were considered exposed; when treatment was initiated during follow-up, the earliest documented ACE inhibitor or ARB initiation date was used. Exposure duration was calculated from treatment initiation to the end of follow-up.*

Abbreviations: *ACE, angiotensin-converting enzyme; ARB, angiotensin receptor blocker; IQR, interquartile range; MACE, major adverse cardiovascular events; MAKE, major adverse kidney events; SGLT2, sodium-glucose cotransporter 2.*

Supplementary Table S2B. Sensitivity Analysis Restricted to Patients Entering the Cohort in 2018 or Later

Outcome	Main analysis, all patients	Cohort entry ≥2018 only
**MACE**		
Patients / events	254 / 52	111 / 13
Events per parameter (EPV)	7.4	1.9
MACE events during SGLT2i exposure	9	5
SGLT2i HR (95% CI)	1.28 (0.55–2.94)	1.14 (0.26–4.90)
p value	0.567	0.861
**MAKE**		
Patients / events	260 / 95	116 / 22
Events per parameter (EPV)	10.6	2.8
MAKE events during SGLT2i exposure	14	7
SGLT2i HR (95% CI)	0.59 (0.31–1.11)	1.04 (0.35–3.07)
p value	0.101	0.940

Note: *The sensitivity analysis was restricted to patients entering the cohort in 2018 or later. Models included the same covariates as Model 2A, except that the cohort-entry-period variable was omitted because all patients belonged to the same calendar-period category. Because of the limited number of events in the restricted cohort, particularly for MACE, these analyses should be interpreted as exploratory and supportive rather than definitive.*
Abbreviations: *CI, confidence interval; EPV, events per parameter; HR, hazard ratio; MACE, major adverse cardiovascular events; MAKE, major adverse kidney events; SGLT2i, sodium-glucose cotransporter 2 inhibitor.*

Supplementary Table S3A. eGFR Slope According to MAKE and MACE Status

Group	n	eGFR slope, median (IQR), mL/min/1.73 m²/year	p value
Overall cohort	263	−2.0 (−4.6 to −0.3)	—
No MACE	210	−2.6 (−4.7 to −0.6)	
MACE	53	−0.6 (−3.2 to 0.7)	**0.003**
No MAKE	166	−1.2 (−3.4 to 0.2)	
MAKE	97	−3.7 (−5.8 to −1.8)	**<0.001**

Note**:** *Individual annual eGFR slopes were estimated using available longitudinal eGFR measurements at baseline and years 1, 2, and 3 of follow-up. Slopes are expressed as mL/min/1.73 m² per year. Comparisons between patients with and without MACE and between patients with and without MAKE were performed using the Mann–Whitney U test.*

Abbreviations: *eGFR, estimated glomerular filtration rate; IQR, interquartile range; MACE, major adverse cardiovascular events; MAKE, major adverse kidney events.*

Supplementary Table S3B. Association of eGFR slope with subsequent outcomes in the 3-year landmark analysis

Outcome	Patients / events	Unadjusted HR (95% CI)	p value	Adjusted HR (95% CI)	p value
MACE	245 / 43	0.95 (0.87–1.03)	0.198	0.94 (0.84–1.05)	0.243
MAKE	258 / 93	1.21 (1.15–1.28)	<0.001	1.29 (1.20–1.39)	<0.001

The 3-year landmark analyses included 245 patients with 43 subsequent MACE events and 258 patients with 93 subsequent MAKE events.. Adjusted models included age, sex, diabetes mellitus, baseline eGFR, albuminuria, and systolic blood pressure; the MACE model was stratified by sex. HRs are expressed per 1 mL/min/1.73 m²/year faster decline in eGFR. The proportional hazards assumption was assessed using Schoenfeld residuals. The global PH test p values were 0.128 for the MACE model and 0.051 for the MAKE model.

Abbreviations: *CI, confidence interval; eGFR, estimated glomerular filtration rate; HR, hazard ratio; MACE, major adverse cardiovascular events; MAKE, major adverse kidney events.*

Supplementary Table S4. Sensitivity Analysis of the Multivariable MACE Model Including Baseline HbA1c

Variable	Primary model HR (95% CI)	p value	HbA1c-adjusted model HR (95% CI)	p value
Age, per 10-year increase	1.52 (1.12–2.06)	0.007	1.53 (1.13–2.08)	0.006
Sex	Stratified	—	Stratified	—
Diabetes mellitus	3.00 (1.60–5.62)	<0.001	2.54 (1.18–5.44)	0.017
Baseline eGFR, per 10 mL/min/1.73 m² increase	0.77 (0.60–1.00)	0.051	0.77 (0.59–1.00)	0.048
log(UACR + 1)	1.07 (0.91–1.26)	0.420	1.06 (0.90–1.25)	0.468
Systolic blood pressure, per 10 mmHg increase	0.97 (0.84–1.11)	0.652	0.96 (0.84–1.11)	0.615
HbA1c, per 1% increase	—	—	1.09 (0.88–1.34)	0.425

Note: *Both models included 254 patients with 52 MACE events. The primary model was stratified by sex. Baseline HbA1c was additionally included in the sensitivity model. The global proportional hazards test p values were 0.074 for the primary model and 0.064 for the HbA1c-adjusted model. All variance inflation factors were <2. The correlation between diabetes mellitus and HbA1c was 0.67.*

Abbreviations: *CI, confidence interval; eGFR, estimated glomerular filtration rate; HbA1c, glycated hemoglobin; HR, hazard ratio; MACE, major adverse cardiovascular events; UACR, urine albumin-to-creatinine ratio.*
